# Circulating Tumor Cell Detection Technologies and Clinical Utility: Challenges and Opportunities

**DOI:** 10.3390/cancers12071930

**Published:** 2020-07-17

**Authors:** Zeina Habli, Walid AlChamaa, Raya Saab, Humam Kadara, Massoud L. Khraiche

**Affiliations:** 1 Neural Engineering and Nanobiosensors Group, Biomedical Engineering Program, Maroun Semaan Faculty of Engineering and Architecture, American University of Beirut, Beirut 1107 2020, Lebanon; zsh11@mail.aub.edu (Z.H.); mma237@mail.aub.edu (W.A.); 2 Department of Pediatric and Adolescent Medicine, American University of Beirut Medical Center, Beirut 1107 2020, Lebanon; rs88@aub.edu.lb; 3 Department of Anatomy, Cell Biology and Physiological Sciences, American University of Beirut, Beirut 1107 2020, Lebanon; 4 Department of Translational Molecular Pathology, The University of Texas MD Anderson Cancer Center, Houston, 77030 TX, USA; hkadara@mdanderson.org

**Keywords:** circulating tumor cells, liquid biopsy, prognosis, cancer management, biosensors

## Abstract

The potential clinical utility of circulating tumor cells (CTCs) in the diagnosis and management of cancer has drawn a lot of attention in the past 10 years. CTCs disseminate from tumors into the bloodstream and are believed to carry vital information about tumor onset, progression, and metastasis. In addition, CTCs reflect different biological aspects of the primary tumor they originate from, mainly in their genetic and protein expression. Moreover, emerging evidence indicates that CTC liquid biopsies can be extended beyond prognostication to pharmacodynamic and predictive biomarkers in cancer patient management. A key challenge in harnessing the clinical potential and utility of CTCs is enumerating and isolating these rare heterogeneous cells from a blood sample while allowing downstream CTC analysis. That being said, there have been serious doubts regarding the potential value of CTCs as clinical biomarkers for cancer due to the low number of promising outcomes in the published results. This review aims to present an overview of the current preclinical CTC detection technologies and the advantages and limitations of each sensing platform, while surveying and analyzing the published evidence of the clinical utility of CTCs.

## 1. Introduction

**Figure 1:**
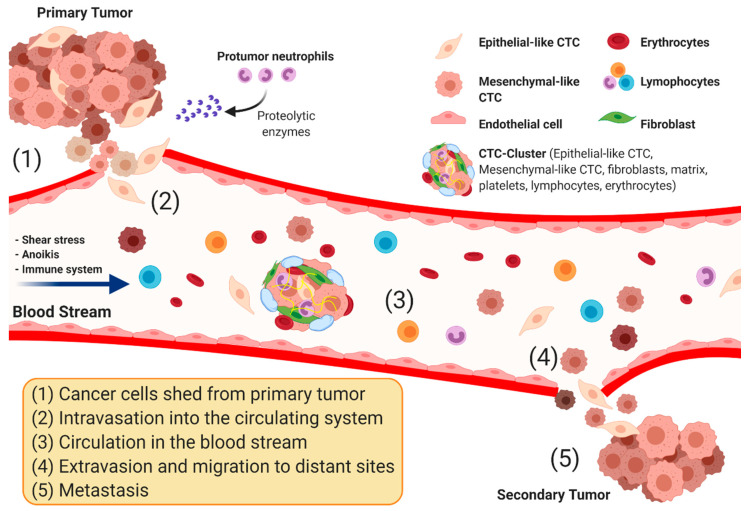
Schematic representation of circulating tumor cells (CTCs) detaching from primary tumor, intravasating into the bloodstream, and circulating to colonize distant organs after extravasating where they create secondary metastasis. Essentially, CTCs undergo epithelial-to-mesenchymal transmission (EMT), where cancerous epithelial cells lose their cell-to-cell contact and develop a more motile and less differentiated mesenchymal phenotype. In addition, infiltrating protumor neutrophils secrete proteolytic enzyme (e.g., matrix metallopeptidase 9 (MMP-9)) to aid CTCs in entering the bloodstream. In the bloodstream, these disseminating cells must overcome blood shear stress, anoikis and immune system response. Once reaching the distant cite, CTCs revert back to their epithelial phenotype and grow into secondary metastasis. CTCs can exist in the form of single cells or cell clusters which has increased metastatic potential. CTCs encompass more of the clonal populations in a tumor, they give a complete picture of tumor composition and how it changes overtime. They can be distinguished from other types of cells circulating in the blood through their differential expression of EMT biomarkers such as epithelial cell adhesion molecules (EpCAM) and Cytokeratin (CK).

The advent of new diagnostic and treatment modalities have improved the 5-year relative survival rate for all types of cancers combined; the survival rate increased substantially from 39% to 70% among white patients and from 27% up to 63% among black patients [1]. It is widely accepted that the capture, enumeration and identification of circulating tumor cells (CTCs) hold significant promise for early cancer screening, diagnosis and prognosis. These cells originate from primary tumors and disseminate to distant sites via the blood (Figure 1) [2,3,4]. The rate of tumor shedding has been estimated to be around 3.2 million cells per gram of tumor per day. The majority of these cells get rapidly cleared, but those that survive bloodstream shear stress and evade recognition by immune cells are speculated to have the highest metastatic potential [5]. Hence, the peripheral blood offers a rich reservoir of cancer-derived materials beneficial for cancer diagnosis and monitoring [6]. Moreover, there is a rich body of literature that supports the diagnostic and prognostic clinical value of CTC analysis in metastatic breast [7], prostate [8], lung [9], and colorectal [10] cancers. In this work, we review the clinical readiness of various attempts to develop, and commercialize, CTC detection technologies, and discuss challenges facing their clinical utility for cancer diagnosis, prognosis, and management.

## 2. Commercialized CTC Detection Technologies

**Figure 2:**
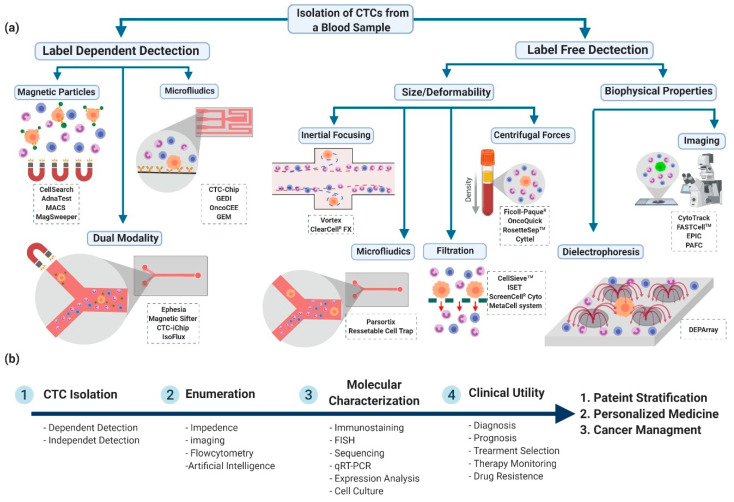
Schematic depicting circulating tumor cells (CTC) detection and isolation technologies. (**a**) Commercialized technologies for CTC detection, enumeration and count. CTCs can be detected based on their molecular or physical properties. Examples of commercial boxes; (**b**) illustration of CTC use as a real-time liquid biopsy after the different isolation approaches for clinical utility.

Several CTC detection platforms have emerged over the past decade, each exploiting a distinctive characteristic of CTCs for sensitive selection and capture. Each technology differs in the biophysical or bimolecular trait leveraged for CTC capture, enrichment, and downstream cellular and molecular characterization, but all of them aim to enumerate CTC and draw clinically relevant conclusions regarding the prevalence of CTC for cancer management. The focus of these methodologies is the detection of CTC clinically, rapidly, and with high sensitivity, selectivity and specificity, while remaining minimally invasive. The differences between CTC and normal blood cells in gene/protein expression, morphology, volume, and biophysical properties had led to the establishment and commercialization of several CTC detection and enumeration devices during the past decade. These commercialized technologies can be categorized based on method of CTC identification as label dependent (affinity-based) or label independent. Then, each category is subdivided into different classes based on its functional detection approach (Figure 2).

### 2.1. Label-Dependent Detection Technologies (Cell Surface Markers)

The most widely used approach for CTC detection and isolation is immune-based detection, whereby antibodies are used to selectively bind cell surface antigens [11]. Tumor cells express different cell surface markers than blood cells and therefore can be separated from the circulatory cells. More specifically, CTC expresses epithelial markers like epithelial cell adhesion molecules (EpCAM) and Cytokeratin (CK) to variable degrees, but not CD45, a differential marker for while blood cells (WBCs) [12]. The antibodies can be conjugated to magnetic nanoparticles or immobilized on the walls of microfluidic chips for CTC capture through the positive selection of CTC or the negative depletion of white blood cells [13,14]. This approach is also termed affinity-based isolation and capture of CTC, and it is the most commonly used technique in Clinical Laboratory Improvement Amendments (CLIA)-certified molecular diagnostics laboratories [15]. A full review of the commercially available CTC label-dependent technologies is summarized in Table 1.

#### 2.1.1. Immuno-Nano and -Magnetic Particles Platforms

One of the leading platforms utilizing label-dependent technology is the CellSearch systems (Veridex), which employ EpCAM-coated ferrofluid nanoparticles for the selection of EpCAM-positive CTCs followed by confirmation with immunostaining for the high expression of CK 8, 18 and the absence of CD45 expression [13]. More than 10 CellSearch kits are available commercially and can identify clinically relevant populations of CTC. One kit received Food and Drug Administration (FDA) approval in 2004, the circulating epithelial cell in vitro diagnostics (IVD) kit [13]. CellSearch has been widely used in monitoring patients with metastatic breast, colorectal, lung or prostate cancer [16]. The first clinical trial with CellSearch was conducted by Cristofanilli and colleagues, where they measured levels of CTCs in metastatic breast cancer; their trial indicated that CTCs could be used as a marker for overall survival (OS) and progression-free survival (PFS) [39]. Ever since, CellSearch technology has proven to be sensitive and reproducible in a large number of studies [40].

Important correlations between CTC count and cancer relapse have already been observed using the CellSearch system, but the technology has some limitations [13]. Firstly, it relies on the expression of EpCAM only and ignores other potential biomarkers. EpCAM has a dynamic expression among CTCs of different origins and at different cancer stages [41]; it becomes highly downregulated when CTCs disseminate from primary sites and undergo epithelial-to-mesenchymal transmission (EMT) to acquire a more aggressive phenotype and seed for metastasis. Studies have shown that EpCAM’s presence has been correlated with localized cancer; however, upon metastasis, its expression, along with that of CK, decreases amid the appearance of mesenchymal markers [2,42]. Secondly, cell isolation through the CellSearch system is followed by cell fixation for stabilization, which prevents further viable cell characterization such as CTC culture, CTC 3D organoid culture or CTC-derived xenografts. Another major pitfall in the CellSearch system is the low purity of captured cells which ranges between 60–70%, the captured CTCs are usually contaminated with blood cells or normal circulating epithelial cells (CEC). In addition, the CellSearch system has a low sensitivity for CTC detection (one cell per 1 mL of a blood sample) [13,16].

AdnaTest^®^ (AdnaGen AG, Langenhagen, Germany) is another commercialized positive selection platform that relies on immunomagnetic beads coated with a cocktail of antibodies for the enhanced capture and enrichment of CTCs in breast, prostate, ovarian and colon cancer [18]. The positively enriched cells are then tested by multiplex RT-PCR for various gene panels (e.g., MUC-1, HER-2 and GA733-2) based on tumor type for CTC validation, an improved design that combines two methodological approaches for enhanced sensitivity and better heterogeneity characterization of CTCs [43]. AdnaTest^®^ offers physicians the opportunity to analyze the clinical relevance of CTCs by testing the gene expression of specific tumor markers in the captured cells and comparing this with their primary and metastatic tumor counterparts, thus possessing a diagnostic and prognostic value on one hand and also allowing the manipulation of treatment regimens. In a clinical study to assess the prognostic validity of CTC in metastatic castration-resistant prostate cancer, AdnaTest detected CTCs in 62% (34/47) of patient samples compared to only 45% with CellSearch (23/47). This difference could be attributed to the fact that AdnaTest identified the CTCs based on the presence of KLK3, PSMA and EGFR transcripts compared to CellSearch, which depends only on EpCAM for capture. In this study, the overall sensitivity of the CellSearch results was considered unfavorable [18]. Although Adnatest combines two approaches in its design, in some cases, CellSearch has superiority because of the technical difficulties accompanied with AdnaTest blood sample processing and preservation. For example, in a comparative metastatic breast cancer clinical study, the CellSearch system was able to detect CTCs in 50% (116/221) of patients compared to AdnaTest, which detected CTCs in 40% of the patients only (88/221). The CTCs detected by AdnaTest had no association with OS nor with PFS in contrast to prognostic outcomes in CellSearch [19].

In addition to CellSearch and AdnaTest, various technologies depend on immunomagnetic beads for CTC enrichment, such as Magnetic-activated Cell Sorting (MACS) systems (Miltenyi Biotec, Bergisch Gladbach, Germany), and MagSweeper (Illumina, San Diego, CA, USA) (reviewed in Table 1). MACS offers both positive enrichment and negative enrichment for the high-efficiency isolation of CTCs. On the other hand, MagSweeper processes larger volumes of blood (9 mL/h) and can detect 1–3 CTCs per 1 mL of unprocessed blood. However, there are unavoidable problems with this approach, the reliance on EpCAM and CK presence and the variation in their expression among the different types of cancer and the different stages is a major drawback for these modalities. In addition, the use of expensive antibodies leads to high detection costs and the internalization of micro- and nanomagnetic affects the viability of captured cells. Moreover, post-capture, CTCs cannot be further analyzed in real-time and live-cell conditions, since the cells are fixed or lysed during the assay process.

#### 2.1.2. Microfluidic Platforms

Microfluidic chips are capable of integrating conventional biological assays at the microscale that can lead to CTC detection, isolation, and even cell culture. Hence, these platforms show a major potential for processing CTCs post-capture and isolation. The precise microfabrication used to build these platforms offer controlled geometries, fluid flow and surface functionalization, leading to directed and regulated cell contact with the chip walls [44]. The relatively high surface areas coated with antibodies provide many sites for CTC capture. The captured cells are fixed on the micro-spot, while remaining blood components are carried away by the flow; this allows nonspecifically bound cells to be washed out, thus ensuring the high enrichment of CTC against leukocytes. The most commonly used antibody is EpCAM, but several chips employ a cocktail of antibodies specific to a particular cancer or use anti-CD45 and/or anti-CD66 antibodies for negative depletion (retaining of leukocytes and eluting CTCs). Today, several devices have been proposed for this functionality, including the geometrically enhanced mixing (GEM) chip, geometrically enhanced differential immunocapture (GEDI) chip, LiquidBiopsy^®^ (Cynvenio Biosystems, Inc., Thousand Oaks, CA, USA), and OncoCEE (cell enrichment and extraction) (Biocept, San Diego, CA, USA). Compared to immunomagnetic bead-based platforms, these devices have higher capture specificity and selectivity, and thus can be used for diagnosis and not only prognosis [17]. A novel surface-based microfluidic capture system for CTC selection with high throughput, high recovery, high purity and full automation is the modular CTC sinusoidal microsystem (BioFluidica, LLC., San Diego, CA, USA). The system is composed of three modules; the first is the selection module, which has 50–320 parallel sinusoidal channels coated with antibodies (anti-EpCAM, and/or anti-seprase/FAP alpha) for CTC capture, the second is the impedance sensor module for label-free counting, and the third is the collection module for collecting captured cells that are stained and imaged consequently. This microsystem can detect up to 50 CTCs/mL with >85% purity [25]. The microfluidic and specifically ligand-based capture approach we discussed allows for the release of cells post-capture for phenotypic processing and molecular analysis. However, some limitations still exist, including limited volume and slow rate processing as well as shear forces that might affect cell viability and cell–ligand attachment if not sufficiently low.

#### 2.1.3. Dual Modality Platforms

This is a new generation of CTC antigen-based capture platforms that combine immunomagnetic beads with the advantages of microfluidics to address the shortcomings of single modality platforms. The CTCs are usually isolated from whole blood via immunomagnetic beads, then passed through microfluidic platforms to isolate the magnetically labeled cells according to size or inertial focusing for higher sensitivity capture (Figure 2a) [44]. A promising example is the CTC-iChip device, which houses three microfluidic components inline for CTC capture. Firstly, the cells are magnetically labeled in whole blood, then the cells are passed sequentially in three microfluidic technologies within a single automated system. The first component is deterministic lateral displacement, which removes nucleated cells from whole blood samples based on cell size; the second component is inertial focusing to line the nucleated cells, and the third component is magnetophoresis to magnetically deflect magnetically labeled cells from unlabeled cells into a separate channel. This platform has a dual format; it either uses positive selection (CTC-iChip^pos^) using EpCAM-labeled magnetic beads or negative selection (CTC-iChip^neg^) using CD44- and CD66b- labeled magnetic beads [37]. In comparison to CellSearch, CTC-iChip^pos^, where CTCs are captured with EpCAM beads and stained for CK, displayed higher sensitivity to the capture of CTC in patients with low CTC count (<30 CTCs/7.5 mL) and achieved a >3.5-log purification (a background of 1500 WBCs/mL of the blood sample) [38]. In a head-to-head comparison with CellSearch in clinical samples with <30 CTCs per 7.5 mL, the number of isolated cells by CTC-iChip^pos^ was significantly higher in 22 out of 36 samples (*p* < 0.001). The isolated cells were then molecularly characterized by RT-PCR, four positive samples were correctly identified and correlated to patients with an EML4–ALK oncogenic fusion protein at their primary tumor, an approach that could not be attained with CellSearch [37]. It is worth mentioning that the CTC-iChip is compatible with standard CTC analysis post-capture protocols and is being developed for diagnosis purposes [38]. With dual modality approaches, the enrichment efficiency is usually higher (>99%) with a higher purity of isolated cells. However, this platform is still immature and not widely investigated. Another promising example is the Ephesia system, which was developed initially to capture leukemic B-cells and then modified to capture CTCs. The sample is made to flow through a diamond-like chip into the capture columns zone. Supermagnetic beads coated with EpCAM antibodies self-assemble into a periodic array under a high magnetic field that creates a dense sieve, which, in turn, captures the passing EpCAM positive cells. This technology avoids the need for costly and complicated microfabrication and can be modified to have different sizes and geometries with varying rates of flow. Ephesia is currently not commercialized but has been shown to be promising for diagnosis, especially with its high capture specificity [33]. In a study comparing Ephesia cell capture technology with the reference CellSearch, CTCs were detected in clinical samples taken from metastatic breast cancer (4/5) or metastatic prostate cancer (6/8) patients. The Ephesia method showed a higher quantity of captured CTC compared to CellSearch in 10 out of the 13 samples [34]. Each capture modality, single or dual, is highlighted in Table 2 with a comparative analysis of their advantages and disadvantages. 

### 2.2. Label-Free Detection Technologies (Cell Surface Markers)

Given that CTCs express EpCAM and CK to varying levels, with some displaying the complete downregulation of these proteins, alternative strategies have been developed and tested to isolate and enumerate CTCs based on their biophysical properties [45]. These platforms discriminate CTCs from other cells based on physical characteristics such as size, density, deformability, and electrical properties. A cellular analysis of CTCs has revealed that they have a greater nucleus-to-cytoplasm ratio, larger sizes (>10 µm compared to <5 µm), a different nuclear morphology, and exhibit different electrical properties than normal cells [46]. In the following section, these differences are highlighted as the detection principle and are summarized in Table 3.

#### 2.2.1. Size-Based Separation

Microfiltration enrichment methods process whole blood through a range of microscale constrictions to capture target cells based on their size or a combination of size and deformability. The recovery efficiency is reduced due to the buildup of filtration resistance resulting from the frictional drag on the blood sample as it passes through the filter, a main limitation of this strategy [42]. 

##### Filter-Based Detection

The most significant physical difference between CTCs and WBCs is size, where CTCs are larger on average. Several platforms aim at sieving CTCs from a blood sample and have been shown to be more selective and efficient than the CellSearch system. These platforms rely on microfiltration, which involves a single membrane with varying pore sizes between 6 and 9 µm and is used to separate CTCs and filter out smaller blood cells. A novel filter-based size exclusion technology called ISET (isolation by size of tumor cells), developed by RareCell Diagnostics, Paris, France, was capable of isolating CTCs independent of their expression of any particular marker. Using this technique, CTCs were detected in patients with hepatocellular carcinoma, breast carcinoma, and melanoma [68,69,70]. In a new study, ISET could detect single CTCs in 80% of 40 evaluated patients with early-stage resectable non-small-cell lung cancer (NSCLC), compared to only 23% of the patients using CellSearch. On the other hand, circulating tumor microemboli (CTM, clusters of ≥3 CTCs) were captured in 80% of patients using ISET but were undetectable by CellSearch. Of the captured single CTCs, 62% were positive for the proliferation marker Ki67, whereas cells within CTMs tested negative [47]. Bobek V. et al. used a new size-based separation modality, the MetaCell^®^ technology, for the enrichment and cultivation of CTCs in vitro. Using this technology, CTCs were detected in 66.7% of patients, with comparable frequencies in patients with operable and inoperable tumors (60% vs. 77.8%). Comparable CTC fractions were observed among patients with metastatic and nonmetastatic tumors (66.7% vs. 66.7%). The CTCs were then cultured in vitro for further downstream applications, thus confirming their viability [46]. Although simple and easy to automate, the filtration system is prone to pore clogging, and requires large volumes of blood. Moreover, the purity of the collected samples has been an issue in some devices [71].

##### Microfluidic Chips

Microfluidic chips have also been developed for CTC size-based detection and termed “three-dimensional microfiltration”, where 3D geometries are constructed and designed to allow the separation of CTCs from background blood components. The Parsortix system is an example of such an approach: it has a stair-like architecture that decreases gradually in width down to 4.5 µm to aid in the capture of larger cells (i.e., CTCs) and provide the necessary physical support above and below the captured cells to prevent morphological changes. CTCs larger than the channel width become trapped in the gaps, while smaller cells pass through. Its design maximizes the length of separation and allows reverse flow for the subsequent release of captured CTCs for downstream interrogation and analysis [72].

##### Centrifugal Forces

Density gradient centrifugation is a typical method for segregating whole blood into its constituents based on differences in sedimentation coefficients. As whole blood is dropped in the liquid gradient while being subjected to centrifugation, cells are distributed along the gradient depending on their density. Erythrocytes or polymorphonuclear leukocytes with lower cellular density are precipitated at the bottom, whereas the heavier mononuclear leukocytes and CTCs remain at the top [73]. Several platforms have been developed for preclinical and clinical applications, the most common being OncoQuick^®^ (Greiner Bio-One International GmbH, Frickenhausen, Germany) and Percoll, Ficoll-Hypaque^®^ (GE Healthcare Life Sciences, Uppsala, Sweden), mostly used in biomedical laboratories to recover peripheral blood mononuclear cells. Despite its long history of use in laboratory environments, there are some drawbacks linked to this technique, mainly the possible loss of CTCs that move either to the plasma region or to the bottom of the density gradient due to the formation of aggregates. It is worth noting that this cell loss could be due to the cytotoxicity of the density medium. Interestingly, the Percoll density (GE Healthcare Life Sciences) gradient medium has some advantages over Ficoll, which include reduced toxicity as well as a wider density gradient range [73,74]. Another platform that uses gradient centrifugation to separate cells is the Cyttel system, which combines anti-CD45 immunohistochemistry and fluorescence in situ hybridization followed by gradient centrifugation and slide smearing to identify CTCs. Tong et al. performed a comparative clinical study between Cyttel method, and the CellSearch platform on 127 patients. The CellSearch system revealed that only 30–50% of lung cancer patients had more than one CTC in 7.5 mL of blood, and just 20–30% of patients had more than two CTCs in 7.5 mL of blood. Using the Cyttel method, CTCs were present in 87% of the patients; among them, 63% had a CTC count of ≥3 cells/3.2 mL, 37% had a CTC count of ≥5 cells/3.2 mL, and 15.2% had a CTC count of ≥8 cells/3.2 mL [75]. 

##### Inertial Focusing

Inertial focusing uses the effects of fluid inertia in microchannels of a certain shape to align microparticles and cells at high flow rates. When randomly dispersed particles, such as cells in blood, flow through a channel with a particle Reynolds number of one or greater, they are subjected to two counteracting inertial lift forces: a force that directs particles toward the channel walls, and another that repels the particles toward the channel centerline. In square or rectangular channels, combining these forces leads to the migration of particles to two to four dynamic equilibrium positions located between the channel centerline and the wall. Following focusing, cells are collected in a smaller volume and significantly concentrated in a size-dependent fashion [76,77]. Sollier E. et al. combined this phenomenon with laminar micro-vortices to trap CTCs until flushed out by perfusion at flow rate; the tailored approach is called Vortex. Their design makes use of multiple expansion-contraction reservoirs placed in series and parallel, which generate multiple vortices when a laminar flow of a sample occurs at a high rate. CTCs are then simply released, after a wash step that removes any remaining small particles, by lowering the flow rate. Using this system, Sollier E. et al. were able to extract and enumerate CTCs from four patients with breast cancer and eight patients with lung cancer. From 7.5 mL of blood, 25–51 and 23–317 CTCs were detected from breast and lung cancer patients, respectively [66].

Another modality that makes use of inertial focusing is the ClearCell FX, developed by Clearbrdige Biomedics. ClearCell FX is a spiral microfluidic device that combines both inertial focusing with the secondary Dean’s flow resulting from curved channels to trap CTCs from a blood sample; this allows for the proper positioning of CTCs within the channel. This modality can process 7.5 mL of blood in less than 10 min but requires red blood cells lysis prior to sample processing. Khoo et al. tested this system on patients with metastatic breast cancer or NSCLC. CTCs were detected in 100% of the patients, with a varied range of CTCs isolated for breast cancer samples (12–1275 CTCs/mL) (Median: 55 CTCs/mL) and NSCLC samples (10–1535 CTCs/mL) (Median: 82 CTCs/mL), respectively [78]. Inertial focusing allows the recovery of viable cells for downstream analysis and does not require complex high-resolution imaging techniques nor the use of expensive antibodies. The emerging evolution of this modality will allow the creation of low-cost CTC detection chips for better cancer management. 

#### 2.2.2. Direct Imaging and Biophysical Properties-based

In addition to size-dependent isolation, other physical traits observed in CTCs are exploited to distinguish them from leukocytes. Two innovative approaches to cell separation are dielectrophoresis and direct imaging, which both depend on cell composition, morphology, and phenotypes. These two platforms are discussed in the following subsections.

##### Direct Imaging

Imaging-based detection makes use of specific fluorescent tags to identify and count CTCs in blood. Several imaging-based CTC detection technologies have been developed and tested and each is unique in its sample preparation, detection algorithm, and fluorophores used. Somlo G. et al. developed a novel fiber optic array scanning technology (FASTcell™) to detect CTCs in patients with locally advanced/inflammatory breast cancer (LABC/IBC), metastatic breast cancer (MBC) and non-small cell lung cancer. The principle of operation of the FASTcell™ technology is based on an array of optical fibers that form a wide collection aperture to allow a wider field of view; this enables the rapid high-fidelity localization of CTCs identified by conventional markers such as CK, DAPI and CD45 without the need for enrichment. The FASTcell™ system can scan a sample of blood on a glass side at a rapid rate of 25 million cells/min, comprising the image resolution and subsequent confirmation of potential detected CTCs. Clinical studies using FASTcell™ have successfully identified CTCs in 62% of LABC/IBC patients, in 82% of MBC patients and 42% of non-small cell lung cancer patients [61,79].

Another novel imaging approach that combines both flow cytometry with fluorescence imaging for high throughput analysis has been developed recently. The technology is referred to as ImageStream, and its detection of CTC depends on the expression of EpCAM, CK, AFP, glypican-3 and DNA-PK together with an analysis of size, morphology and DNA content. In a clinical study of patients with hepatocellular carcinoma (HCC), between one and 1642 CTCs were detected in the blood samples of HCC patients (45/69) compared to zero CTCs in the controls (0/31) [63]. In a comparative study between CellSearch and ImageStream, the number of counted CTCs from a reference value did not differ significantly between both methods, however ImageStream had a lower level of precision when fewer CTCs were analyzed. ImageStream, if developed properly and tested extensively, can serve as a platform for CTC enumeration for early diagnosis [80].

##### Dielectrophoresis

Dielectrophoresis (DEP) is a liquid biopsy separation method that relies on particles with different polarization that move differently under a nonuniform electric field [81]. Microchips that use the DEP technique have been developed for isolating and capturing CTC via multiple integrated electrodes, generating a nonuniform alternating electric field [42,82]. The Apostream™ system by ApoCell was the first commercially available DEP field flow fractionation where CTCs and leukocytes are separated based on the differences of their conductivities. Its methodology requires an initial enrichment step. The recovery rate recorded is over 70%, the viability is higher than 97% and the processing time is 1 h for a 10 mL blood sample; however, the purity obtained is less than 1% [65]. DEPArray, on the other hand, is the second commercialized DEP strategy to separate CTCs in a blood sample using controllable electrodes. This platform is designed for single CTC capture, allowing downstream gene analysis and sequencing [64]. In spite of the advantages demonstrated by DEP-based detection and capture methods, there are some limitations. Among these are the low sample volumes and the varying dielectric characteristics of cells due to ion leakage; this limits the isolation time. In addition, the electric resistance of the running medium used must be low, which is not achievable for all samples studied, especially those from diabetic patients [42].

As in label-based enrichment technology, label-free enrichment methodologies have their own advantages and disadvantages that are presented in Table 4. 

## 3. CTC Clinical Utility: Reporting Capabilities

In 2015, a search for “circulating tumor cells” in the “ClinicalTrials.gov” website revealed 296 studies involved in CTC detection and capture in patients with metastatic disease [83]. Currently, in 2020, the same search engine showed 867 studies on CTCs registered in the U.S. National Library of Medicine website, 271 of which are complete, leaving 596 studies in progress. Of these completed studies, only 60 have published their results with promising outcomes. The fact that the number of clinical studies involving CTCs has doubled in less than four years highlights the clinical and commercial interest in their potential. On the other hand, the low number of promising results point to serious challenges facing the clinical utility of CTC detection technology. The latter includes demonstrating high accuracy in capturing viable CTCs discussed in the previous sections and predicting clinical outcomes of therapeutic regimens which we will discuss in this section. 

Among the clinical uses of CTC detection is cancer prognosis, which is significant for clinical decision making as prognostic estimation is useful for assessing the risks and benefits of the proposed treatment. Huge efforts have been made to understand the clinical utility of CTCs to predict prognosis and guide therapeutic decisions. We selected seventeen studies to demonstrate and confirm the prognostic, and sometimes the diagnostic, implications of CTCs in various types of cancer using different CTC detection technologies including CellSearch. In these studies, like in most cases, patients who had a higher count of CTCs (unfavorable count) have had a worse prognosis, measured primarily by PFS and OS survival estimates. The results of these trials shed light on the real possibilities of CTC counting and gave deeper insights on the potential of using CTCs as liquid biopsies (Table 5, Figure 2b).

### 3.1. Prognosis: Pretreatment Staging Assesmnet

The pretreatment staging of patients with cancer and risk stratification based on clinical and histopathological findings greatly improved prognostication and treatment allocation for many cancer subtypes; however, there remains much room for improvement [99]. Evidence show that the implementation of CTC detection and quantification can improve the accuracy of patient preoperative staging [100]. In a recent study conducted on 100 preoperative patients with esophageal cancer, CTC detection using the CellSearch system was correlated with clinicopathological parameters for measuring the prognostic outcome. CTCs were detected in 18% of all eligible patients. Compared to the histopathologic stage (invasion, tumor grade and histological subtype), CTC positive patients had inferior OS (Hazard Ratio [62]: 3.128, *p* < 0.001) and relapse-free survival (RFS) (HR: 5.063, *p* < 0.001) compared to CTC negative patients. Even in patients with non-metastatic tumors and lymph node invasion, CTC presence indicated worse OS and RFS. A multivariate analysis identified CTCs as strong independent prognostic indicators of tumor recurrence (HR: 5.063, *p* < 0.001). The outcome of this study suggests the clinical relevance of CTCs as preoperative prognostic and staging parameters in esophageal cancer [93]. In a similar study with CellSearch, CTC detection in preoperative patients with colorectal cancer (stages I–IV) has been used as a prognostic marker with a threshold of at least one CTC per 7.5 mL. CTC detection was more frequent in patients with metastatic compared to those with non-metastatic disease. Patients with non-metastatic CTC positive disease have a worse OS (38.4 months) compared to CTC-negative patients (49.8 months) in the same cohort (*p* < 0.001). Importantly, multivariate analysis revealed that CTC detection was the strongest prognostic predictor independent of other clinicopathological parameters; in fact, there was no association between primary tumor characteristics or clinicopathological parameters (age, sex, disease site, T stage, N stage, resection margins and distribution of metastasis) and CTC detection in non-metastatic patients [92]. These studies provide evidence-based utility of CTCs as predictive biomarkers which can be further used to stratify patients’ risks within the different stages of the disease. However, the use of CellSearch only investigates a subpopulation of CTCs (EpCAM and CK positive), ignoring a broader spectrum of cells without enumeration or analysis. In addition, such studies must be validated in further cohort forms in multi-institutional trials with longer follow-up times in order to evidentially conclude whether CTC detection should be included in prognostic measures and treatment guidelines.

### 3.2. Prognosis: Response to Therapy

The prognostic significance of CTC is also true for patients undergoing a new line of treatment. Using the CellSearch platform, predictive outcome measures of CTC count and chromogranin A (CgA) were investigated in 138 patients with metastatic neuroendocrine neoplasms receiving a new line of treatment (somatostatin analogues, chemotherapy, PRRT, and TAE). Of all patients, 51% had received previous anticancer therapy, 41% were receiving long-term SST (somatostatin analogue), and 60% of patients tested positive for CTC (at least one CTC detected). Fifteen weeks after starting the new line of therapy, patients with zero CTC count had the highest OS (49.1 months), patients with a CTC count from one to eight had lower OS (21.5 months, 95% CI: 1.20–5.16), and patients with a CTC count above eight had the worst OS (13.3 months, 95% CI: 2.70–9.74). The changes in CTC count significantly correlated with the response to treatment and OS, suggesting their potential use as surrogate markers to direct clinical decision-making. On the other hand, changes in CgA were not significantly associated with survival [85]. 

In another study, a pooled analysis of 1944 patients with metastatic breast cancer from 20 different studies at 17 European centers validated the prognostic clinical utility of CTCs. Before using a new line of treatment, 47% of patients tested positive for CTCs using the CellSearch system (threshold ≥ 5CTCs/7.5 mL). Patients with elevated CTCs at baseline had an inferior OS (HR: 2.27, *p* < 0.0001) and PFS (HR: 1.92, *p* < 0.0001) compared to patients with a CTC count lower than five CTCs/7.5 mL. After the new line of treatment, any increase in CTC count correlated with shortened OS and PFS. When the CTC count was added to the full clinicopathologic predictive models, the prognosis accuracy was improved according to using the likelihood ratio (LR) χ^2^ statistical analysis. It is worth mentioning that serum tumor markers (CEA and CA15-3) did not show any significant prognostic value, even when added to the clinicopathologic predictive model [7]. However, the surrogacy of CTC count as a method to manage treatment was not assessable in this study because the data was not collected from randomized therapeutic trials. In other words, assessing switching treatments based on CTC count was not evident in assessing the response to treatment. Similar patterns were obtained in other studies listed in Table 5, all of which confirm that elevated levels of CTC are clinically important markers that reflect an unfavorable tumor response to treatment and worse patient outcomes.

### 3.3. Beyond Diagnosis and Prognosis: Molecular Characterization and Treatment Decisions

Beyond diagnosis and guiding prognosis when enumerated, CTCs have been used as pharmacodynamic biomarkers to assess inherited or acquired resistance in response to specific treatment regimens. The molecular characterization of CTC has influenced the choice of therapeutic options in some cases. It also has improved our understanding of the underlying mechanisms of cancer metastasis and helped in discovering new targets for therapeutic manipulation. For example, in a phase I clinical trial, CTCs were assessed based on their count and the expression of insulin-like growth factor-1 receptor (IGF-1R) to determine therapeutic approaches. The CellSearch system was used to count CTCs expressing IGF-1R in patients treated with monoclonal antibodies against IGF-1R, either alone or in combination with docetaxel. Out of 26 patients, 23 had positive IGF-1R CTCs and had responded better to the combinatorial treatment compared to the remaining three patients whose CTCs were negative for IGF-1R. Such a study suggests the potential use of CTCs as predictive markers for the choice of administered chemotherapy [101]. Another example is a cross-sectional cohort study carried by Scher and colleagues on 193 patients with mCRPC; the EPIC platform was used to correlate the expression and localization of the nuclear androgen-receptor splice variant 7 (AR-V7) in CTCs in response to taxanes and androgen receptor-signaling inhibitor (ARSI). Overall, patients with nuclear AR-V7 positive CTCs had a poor prostate-specific antigen response, and a short PFS and OS when treated with ARSI, while those treated with taxanes had a much lower risk of death compared to the first group [62].

Several clinical studies have reported the use of CTC with circulating tumor DNA (ctDNA), tumor-derived DNA released into the blood via apoptosis or the necrosis of shedding cells from primary and metastatic lesions, as a complementary biomarker for treatment assessment. In a phase II clinical trial of erlotinib and pertuzumab in patients with advanced NSCLC, a decrease in CTC count upon treatment was correlated with longer PFS. In addition, patients with EGFR mutations showed a substantial reduction in CTC count throughout treatment. The mutational analysis of EGFR showed that ctDNA had higher sensitivity in detecting mutations compared to CTC and, upon treatment, a decrease in mutational load suggested a partial response to treatment. This study, and another mentioned in Table 5, suggest the potential role of CTC combined with ctDNA as biomarkers for the early indication of treatment response [98]. 

## 4. Discussion

In the past two decades, our improved understanding of the biology of CTCs and their role in cancer metastasis has opened the door to a myriad of technologies aimed at exploring the clinical potential of CTCs as biomarkers for cancer. CellSearch remains the only successful platform to obtain FDA approval, while many are still in preclinical and clinical trial stages. Antigen-free approaches are showing a lot of potential for clinical success as they overcome the heterogeneous expression of expressed membrane proteins in CTCs. Affinity-based detection platforms are primarily designed to detect, and count CTCs based on the expression of epithelial markers, mainly EpCAM and CK, but such markers are proven to be downregulated upon undergoing EMT. Such inadequacy necessitates either finding a universal selection epithelial and mesenchymal cell surface marker to prevent cross-reactivity with other types of cells in the blood or adopting physical, mechanical and electrical properties as selection markers for isolating CTCs from their surroundings. In addition, any selection approach also may need to distinguish single cells and clusters (few currently do, see Table 1). The presence of the latter has been linked with a poor prognosis [102].

Owing to CTC heterogeneity and the different approaches used to assess the sensitivity and specificity of each detection platform, as highlighted in Table 1 and Table 3, it is often hard to comparatively evaluate the output of these different platforms in diagnosis, prognosis, and cancer screening. The interpretation of such metrics is quite challenging because of some major limiting factors such as the volume of blood used, the size of the cancer patient population, the used mathematical model for evaluation and, most importantly, the reference for comparison (i.e., CellSearch, blood sample, other microdevices, etc.). Several clinical studies have highlighted the prospects for monitoring cancer patients, while others have used CTC to dissect its biological metastatic potential. One of the exciting clinical applications for CTC technology is correlating CTC counts with clinical outcome measures such as OS and PFS. Most published studies showed that CTC enumeration was the strongest prognostic biomarker for patients’ survival rates, even with multivariate analysis [85,92,93,95].

On the other hand, despite the few promising results obtained with CTC enumeration and molecular characterization for prognostic predictions and treatment response, implementing CTC count as a method to manage treatment in cancer patients is not attainable. In a cohort clinical trial (SWOG S0500) in patients with metastatic breast cancer and no CTC decrease in response to the first-line chemotherapy, switching to second-line chemotherapy did not affect CTC count, nor did it improve OS [103]. This means that although an elevated CTC count is correlated with ineffective treatment response, changing the treatment while considering CTC count only might not be beneficial. The clinical utility of CTCs needs to be validated through randomized clinical interventions in which therapy decisions are based on CTC analysis and established endpoints. Additionally, comparing the outcome of the different CTC clinical studies is challenging due to the use of different patient populations, different CTC detection technologies, different definitions of threshold positivity, different statistical methodologies, and different clinical endpoints used for outcome reporting. To date, standardized methods for clinical CTC isolation and identification are still lacking, contributing to controversial outcome measures in some cases. In addition, the heterogeneity and rarity of CTCs, along with the relatively small cohort of patients tested, might influence the findings and interpretations of the various prognostic and diagnostic outcome measures being tested. With ambiguities in the clinical relevance of CTC, hardly any CTC detection technology is going to be introduced and implemented in routine clinical practices any time soon. Thus, a shift from just counting CTC into a more detailed molecular and functional characterization of CTC will certainly provide a comprehensive picture of the malignancy using only patient blood. In addition, complementing CTC analysis with other liquid biopsy biomarkers such as circulating tumor DNA (ctDNA) can enable inclusive results to better understand the tumor status of a patient [104,105]. The fact that ctDNAphylogenetic analysis can depict early stages of cancer evolution [106] and elucidate the therapeutic resistance of tumors to chemotherapy through genetic mutations [107] suggests its clinical importance. Such combination studies using two liquid biopsies are promising, but the performance characteristics have been modest and require further investigation.

## 5. Conclusions and Future Prospects

Despite all the advances in CTC detection technologies and their diverse capture and enrichment systems, many significant challenges are yet to be met, particularly those with respect to analytical and clinical sensitivities. Adopting such tools into routine clinical practice will demand laborious studies into their analytical validity, clinical validity and clinical utility. These tools, coupled with bioinformatics tools and annotated databases, will provide evidence as to whether detected genomic aberrations in blood may aid in predicting the most suitable cancer therapy on a personalized level.

## Figures and Tables

**Table 1:** Commercially available platforms for CTC detections based on their cell surface markers. (label-dependent technologies).

Device	Technology	Tumor Type	Clinical Value	Remarks	Ref.
**Magnetic Nanoparticles**					
CELLSEARCH^®^ (Janssen Diagnostics)	EpCAM-coated ferrofluid nanoparticles for the selection of EpCAM+ cells. The captured cells are then confirmed by IF staining of CK 8, 18,19 and the lack of CD45	-Metastatic breast cancer -Colorectal -Prostate	-Prognosis -Treatment	-FDA approved -use of antibodies (markers dependence) -Low purity of captured CTC -Sensitivity: 27%, 32%, 70% -Specificity: 89%, 99.7%, 93% -Most clinically validated capture technique	[16,17]
AdnaTest (Adnagen)	Magnetic beads coated with a cocktail of antibodies (EpCAM, MUC-1, etc.). Captured CTC are then analyzed by multiplex RT-PCR gene panels	-Breast -Prostate -Ovarian -Colon	-Prognosis -Treatment regimen	-Analyzes blood and bone marrow samples -Downstream RNA analysis post enrichment by RT-PCR -High sensitivity -High contamination with WBCs -Detection limit: >2 CTCs/7.5 mL ^a^ -Sensitivity: 73% ^a^	[18,19]
MACS system (Miltenyi Biotec)	Immunomagnetic CTC enrichment by antibodies against cell surface markers or by an intracellular anti-pan CK antibody	-Non-small-cell lung cancer (NSCLC) -Breast (HER2+)	-Prognosis	Identifies EpCAM negative CTCs but not CK negative ones Can work with leukocytes depletion (negative enrichment by anti CD45)	[20,21]
MagSweeper (Illumina)	Immunomagnetic isolation of CTC by antibodies against EpCAM and cell surface markers from unfractionated blood samples	-Breast -Prostate -Colorectal	-Genetic profiling -Drug discovery	-High purity of captured CTC (almost 100%) -High throughput processing (9mL/hr) -Detects 1–3 CTCs/mL -Sensitivity: 100% ^b^	[17,22]
**In vivo**					
GILUPI CellCollector™	EpCAM-coated wire placed intravenously in patients for CTC collection	-Breast	-Detection	-In vivo based-detection of CTC -Processes large volumes of blood -Invasive-Time consuming	[23]
**Microfluidics**					
Modular Sinusoidal Microsystems (BioFluidica)	Combination of three modules for CTC selection, counting, and enumerating. The chip consists of 320 sinusoidal microchannels coated with antibodies for the capture of CTC followed by phenotypic identification.	-Pancreatic	-Diagnosis	-Electrical sensor for counting and determining the viability -Cell enumeration is based on impedance sensor -High purity (>86%) with an excellent yield of recovered -Processes 7.5 mL/h	[24,25]
GEDI	Geometrically enhanced differential immunocapture where antibodies against HER2 and PSMA are immobilized with high capture specificity from unprocessed blood	-Breast -Prostate	-Treatment regimen -Correlation between CTC and primary tumor	-High capture specificity -Detects up to 27 CTCs/mL -high purity (around 26%) -Sensitivity: 94% ^a^	[26]
Herringbone (HB) Chip	High throughput microfluidic mixing device that allows efficient capture of CTC on antibody-coated surfaces	-Prostate	-Histological analysis	-Minimal studies have been assessed and all are preclinical -Processes 4.8 mL/h -Can detect up to 12 CTCs/mL -Purity of captured CTC is around 14%	[27,28]
GEM chip	Geometrically Enhanced Mixing chip structure that allows enhanced capture of CTC on antibody-coated surfaces	-Pancreatic	-Monitoring treatment response -Diagnosis	-Uses of antibodies or cocktail of antibodies -High selection efficiency -Processes 3.6 mL/h	[29]
OncoCEE (cell enrichment and extraction) (Biocept)	Microfluidic chip with internal surfaces functionalized with a cocktail of antibodies against biotumor-associated markers and mesenchymal markers.	-Breast	-Prognosis -Diagnosis -Treatment regiment	-High probability of CTC capture -Analysis of CK+ and CK− CTC is feasible -Sensitivity: 95% ^b^ -Specificity: 92% ^b^	[30]
LiquidBiopsy^®^ (Cynvenio)	Microfluidic chip with functionalized surfaces (coated with antibodies) for positive selection of CTC with direct automated DNA analysis	-Breast -Lung	-Genetic profiling of CTC	-Processes 5mL/h -20% error accuracy and 25% error precision -High purity of detected cells -Sheath flow decreases non-specific binding	[31]
Graphene oxide (GO) Chip	Nanosheets of graphene oxides functionalized with capture antibodies against cell surface markers of CTC with high sensitivity	-Breast -Pancreatic -Lung	-Prognosis	-High capture yield -Processes 1–3 mL/h -Minimal studies have been assessed and all are preclinical -Sensitivity: 73 ± 32.4 at 3–5 cells per mL of blood ^c^	[32]
**Dual modality**					
Ephesia (CTC-chip)	Micromagnetic particles functionalized with EpCAM antibodies are self-assembled in a microfluidic platform (columns)	-Breast -NSCLC -Prostate -Colorectal	-Prognosis -Diagnosis	-High capture specificity -Processes more than 3 mL/h -Viability of captured cells maintained at 98% -Sensitivity: 99.1% ^c^ -Specificity: 100% ^c^	[33,34]
IsoFlux (Fluxion)	A microfluidic platform of controlled flow and immunomagnetic capture bead system	-Breast -Prostate	-Diagnosis	-50% rate of capture -Capacity to detect genetic alterations	[35]
Quadrupole magnetic separator	Negative CTC enrichment by combining viscous flow stress and magnetic force for the recovery of unlabeled CTC	-Breast	-Prognosis -Diagnosis -Treatment selection	-Detects heterogeneity among CTC by IF -Multiparameter analysis is required -Minimal studies have been assessed and all are preclinical	[36]
CTC-iChip	Deterministic lateral displacement, inertial focusing, and magnetophoresis for rapid isolation of leukocytes using anti CD45 and anti CD66B antibodies (negative enrichment) or EpCAM activated beads for CTC enrichment (positive enrichment)	-EpCAM positive cancer -EpCAM negative cancer	-Diagnosis	-Developed at Janssen Diagnostics (in progress) -Positive and negative enrichment -Combines size-based separation of WBCs -Processes 8 mL/h -Low purity of captured CTC (around 8%) -Detection limit: <30 CTCs/7.5 mL ^d^	[37,38]

^a^ Positive agreement between the device and the CellSearch system results; ^b^ positive agreement between test results and blood origin (blood samples from known metastatic cancer patients are true positives, while blood samples from healthy subjects are true negatives); ^c^ positive agreement with immunofluorescent staining for CK and imaging; ^d^ positive agreement with blood samples from healthy subjects and digital RNA-based PCR (dPCR) for RNA-based signature detection.

**Table 2:** Major advantages and disadvantages of CTC labeled detection methods.

Method	Advantages	Disadvantages
Magnetic Nanoparticles	Fast Multiplexed processing High capture and enrichment efficiency	Expensive Low cell viability Dependence on expressed proteins Hard to automate
Microfluidic chip	Minimal sample preparation Less sample and reagent demand Cheap High sensitivity and efficiently High cell viability	Limited sample volume Slow flow rate
Dual Modality	Very high enrichment efficiency High purity	Expensive Poorly investigated technology Moderate sensitivity

**Table 3:** Commercially available platforms for CTC detections based on their biophysical properties (label-free technologies).

Device	Technology	Tumor Type	Clinical Value	Remarks	Ref.
**Filtration**					
ISET^®^ (Rarecells Diagnostics)	Filter based isolation and enrichment (PCL based filters)	-Lung (NSCLC) -Breast -Melanoma -Hepatocellular carcinoma	-Prognosis -Treatment regiment	-High efficiency capture compared to CellSearch -Label-free (no need to use antibodies) -Detection limit: 1 CTC/mL -Sensitivity: 76.37% ^a^ -Specificity: 82.39% ^a^	[47,48]
MetaCell^®^ system (MetaCell Ltd.)	size-based enrichment and separation	-Esophageal -Lung -Pancreatic	-Diagnosis -Prognosis	-Allows post-capture analysis and cell culture	[46]
Parylene filter (Circulogix)	Filter based isolation and enrichment	-Breast	-Diagnosis -Prognosis	-Post-capture downstream analysis for enumeration and immunophenotypic characterization -Fixation prior to capture eliminates post capture functional assays (cell culture and protein extraction and analysis) -Studies have been assessed in preclinical setups -Detection limit: 25 CTCs/7.5 mL -Capture efficiency: ~90%	[49]
ScreenCell^®^ Cyto	Filter based size-exclusion separation and enrichment of CTC	-Melanoma	-Diagnostics -Treatment regimen (personalized medicine)	-Post-capture analysis and cell culture -Allows microscopic examinations of collected CTC -Minimal studies have been assessed and all are preclinical	[50]
CellSieve (Creatv MicroTech)	micofilter based isolation and enrichment	-Breast -Prostate	-Prognosis -Diagnosis	-High efficiency isolation compared to CellSearch technology -Post capture histo- and immune-phenotypic characterization of CTC	[51]
**Microfluidics**					
Parsortix™ technology (Angle plc)	Microfluidic separation of CTC based on their size and deformability. Viable cells are released by reversing the flow.	-Ovarian	-Diagnosis	-Antigen-independent capture with subsequent molecular analysis -Minimal studies have been assessed and all are preclinical -Sensitivity: 92% ^b^ (in primary and relapse ovarian cancer) -Specificity: 100% ^b^	[52]
**Density gradient separation**					
RosetteSep™ CTC Enrichment Cocktail/EasyStep CD45 Depletion (STEMCELL Technologies)	Immunodensity negative selection for CTC using tetrameric antibody complexes that recognizes CD45, CD66b and glycophorin on WBC and RBC	-Pancreatic -Breast	-Prognosis	Unwanted cells are targeted for removal with Tetrameric Antibody Complexes that pellets with RBCs	[53]
OncoQuick(Greiner BioOne, Frickenhausen, Germany)	Separation of erythrocytes and some leukocytes from CTC by porous membrane filtration followed by density-grade centrifugation for CTC enrichment	-Gastrointestinal cancer -Advanced breast cancer	-prognosis	-Dual technology for separation of CTC based on size and buoyant density -High tumor cell rate recovery compared to other density-gradient techniques -Post-CTC capture processing is possible	[54,55]
Cyttel	Negative immune-magnetic selection of WBC (CD45 antibody) followed by gradient centrifugation and slide smearing of isolated CTC	-Lung (NSCLC)	-Prognosis -Treatment regimen	-High detection rate (bimodal identification of CTC: negative selection followed by in situ hybridization)	[56]
AccuCyte–CyteFinder (RareCyte)	Automated rapid imaging of single rare cells, CTC in this case, preceded by density-based cell separation	-adenocarcinoma	-prognosis	-Dual technology platform for single-cell analysis -high sensitivity detection of CD positive CTC -ability to analyze RNA post capture and enrichment -minimal studies have been assessed and all are preclinical	[57]
**Functional Assays**					
EPISPOT	Negative enrichment using CD45 depletion and short-term culture	-Breast	-Prognosis	-Allows CTC detection based on protein secretion -High sensitivity and specificity -Independent of tumor antigen phenotype capture -Allows quantification of CTC	[58]
Vita-Assay (Vitatex)	Functional cell separation using density gradient centrifugation followed by preferential adhesion of CTC to collagen adhesion matrix (CAM-enrichment).	-Prostate	-N.A.	-Allows CTC detection based on invasion properties -Low purity (0.5–35%)	[59]
**Imaging**					
CytoTrack	The blood sample is speed on a glass disc that is rotated at high speed. Fluorescently labelled cells against EpCAM are scanned with laser beam	-Breast	-Diagnosis	-Analyzes 100M cells/min -Low recovery rates of CTC	[60]
FASTcell (SRI)	Fiber optic array scanning technology (FAST)	-Breast	-Prognosis -Guided therapy (personalized treatments)	-High sensitivity of CTC detection based on biomarkers expression -Allows simultaneous detection of multiple tumor specific biomarkers -Analyzes 25M cells/min	[61]
Epic (Epic Sciences)	RBC lysis and IF for CK, CD45, and DAPI and other markers followed by high-definition imaging	-Prostate	-prognosis -treatment regimen	-Unbiased screen of all blood nucleated cells for detection of individual CTCs and clusters	[62]
ImageStream^®^ (Amnis)	Immunogenetic sorting of blood followed by flow cytometery and fluorescent microscopy for CTC enumeration.	-Hepatocellular carcinoma	-Diagnosis	-Low precision when CTC count is low -Analyzes 5000 cells/s -Sensitivity: 68.75% ^c^ -Specificity: 72.97% ^c^ with likelihood ratio of 2.544	[63]
**Dielectrophoresis**					
DEPArray™ (Silicon Biosystems)	Moving dielectrophoretic cages for cell capture coupled with sanger sequencing	-Breast	-Tumor and treatment monitoring -Prognosis	Isolation of single CTCs for dowstream gene analysis	[64]
ApoStream^®^ (ApoCell)	Detection of CTC based on dielectrophoric Field-flow Fractionation (DEP-FFF) in a microfluidic chamber	Breast	-N.A.	-Detection independent of EpCAM expression; useful for viability analysis and culture -processes more than 10 mL/h -Studies have been assessed in preclinical setups	[65]
**Inertial focusing**					
Vortex	CTC extraction using microscale vortices and inertial focusing	-Breast -Lung	-Prognosis -Diagnosis -Treatment regimen	-Fast processing time of samples (20 min per 7.5 mL of blood) -High CTC integrity and purity post-detection (>50% up to 94%)	[66]
ClearCell^®^ FX (Clearbridge BioMedics)	Separation of CTC based on size using Dean Flow Fractionation (DFF) (inertial focusing)	-Lung	-Molecular diagnostics	-Captured CTC can be analyzed post-capture and enrichment in culture -Low recovery rates -Label free (no need for antibody use) -Single step isolation and retrieval process from any type of body fluids -Processes 1–1.5 mL/min -Sensitivity: 80.4% ^c^ -Specificity: 85.7% ^c^	[67]

N.A.: not available; ^a^ Agreement with presence of tumor biomarkers (i.e., CEA, NSE, Cyfra21-1) using ELISA; ^b^ Utilizing a cut-off threshold value for the expression of 30 genes to retain 100% specificity; ^c^ Obtained by ROC curve analysis (cut-off = 3.5 CTCs).

**Table 4:** Major advantages and disadvantages of CTC label-free detection methods.

Method	Advantages	Disadvantages
Microfiltration	Rapid processing of large volumes High efficiency	Low purity Membrane clogging Different size of CTC Difficult to detach CTC from the filter
Density gradient centrifugation	Inexpensive Reliable	Loss of large CTC and cell aggregates Low purity Additional enrichment techniques required
Inertial Focusing	Precise Fast sample processing (minimal time) Simple structure High throughput Freedom of external field	Complicated principle Morphological deformation of captured cells
Direct Imaging	High resolution identification of captured cells	Difficult sample processing Loss of cells under investigation
Dielectrophoresis	Single-cell isolation High cell viability High efficiency	Limited volume Low purity in some devices Cell electrical properties can be affected during the procedure Large number of parameters must be controlled simultaneously

**Table 5:** Selected CTC clinical studies on outcome measures after the year 2012.

Technology	Cancer (Subtype)	Clinical Utility	Number of Patients	Clinical Scenario	Outcome Measure	Remarks	Ref.
Cell Adhesion Matrix (CAM)-initiated CTC enrichment and flowcytometry	Advanced Epithelial Ovarian Cancer (stages I–IV)	Diagnosis Prognosis	129	-Invasive subpopulation of CTC (bind to collagen matrix type I & verified by flow cytometry). -iCTC threshold >5 CTCs/1 mL	OS and PFS	-88.6% had iCTC > 5/1 mL -More iCTCs in higher stage disease (38.5% in stage I vs. 95.2% in stage IV patients) -iCTC above threshold correlated with inferior OS and PFS -iCTC correlated better with OS and PFS compared to CA125	[84]
CellSearch	Metastatic breast cancer	Prognosis	Pooled analysis of 1944 patients from 20 studies	-New line of treatment -CTC threshold ≥5 CTCs/7.5 mL	OS and PFS	-46.9% patients had ≥5 CTCs/7.5 mL -CTCs ≥ 5/7.5 mL had worse OS (HR:2.78) & PFS (HR:1.92) -An increase in CTC count post treatment correlated with decreased OS and PFS	[7]
CellSearch	Metastatic neuro-endocrine neoplasms	Prognosis	138	-New line of treatment -CTC threshold in 3 groups (1, 1–8, >8 /7.5mL)	OS and PFS	10–15 weeks post treatment: strong association between CTC count and OS and PFS	[85]
CellSearch	Metastatic neuro-endocrine tumors	Prognosis	176	-CTC threshold ≥1 CTCs/7.5 mL	OS and PSF	-49% of patients had ≥1 CTC/7.5 mL -CTCs ≥ 1/7.5 mL was associated with inferior OS (HR: 8) and PFS (HR: 6.6) -holds in multivariate analysis	[86]
CellSearch	Non-Metastatic breast cancer (stages I–III)	Diagnosis Prognosis	Pooled analysis from 3173 patients	-CTC threshold ≥1 CTCs/7.5 mL	DFS, breast cancer-specific survival, OS	-CTCs in 20.2% of patients -presence of CTC associated with unfavorable clinical features -CTCs ≥ 1/7.5 mL associated with inferior DFS (HR: 1.82), distant-DFS (HR: 1.89), breast cancer specific survival (HR: 2.04), OS (HR: 1.97)	[87]
CellSearch	Non-Small-Cell Lung Cancer	Prognosis	97	-Before and after one cycle of chemotherapy -CTC threshold ≥50 CTCs/7.5 mL	OS and PFS	-85% tested positive for CTC -Before one cycle of chemotherapy: * CTCs ≥ 50/7.5 mL was associated with significantly worse OS & PFS -After one cycle of chemotherapy: * CTCs < 50/7.5 mL was associated with similar OS but better PFS	[88]
CellSearch and RT-PCR among others	Non-Small-Cell Lung Cancer	-Diagnosis -Prognosis	Pooled analysis from 1576 patients from 20 studies	-CTC threshold ≥1 CTCs/7.5 mL	OS and PFS	-CTC was correlated positively with lymph node metastasis (OR = 2.06) and tumor stage (OR = 1.95). -CTC were associated with shorter OS (RR = 2.19) and PFS (RR = 2.14) indicating poor prognosis	[89]
ScreenCell Cyto filtration device (size-based isolation)	Lung cancer (majority were stage I and II)	-Diagnosis	77	-Differentiate benign from malignant lesions using CTC count as well as clinicopathologic and histologic features	Diagnosis	-CTCs divided into 3 groups: Malignant features (MG), Undefined Malignant features (UMF) and benign features (BF). -CTC-MF count correlated with tumor size and stage with high sensitivity and specificity. -CTC-UMF were detected in 8% of malignant patients and 5% of benign patients -CTC-BF were detected in 88% of benign patients and 1% in malignant patients	[90]
CellSearch	Newly diagnosed breast cancer	-Diagnosis -Prognosis	404	-CTC assessment before undergoing surgical treatment -CTC threshold ≥1 CTCs/30 mL	Breast cancer-related death (BRD) and Relapse-free survival (RFS) at 4-years	-CTCs were detected more frequently in high stage tumors: * 15% of benign tumor patients * 19% in DCIS patients * 16% in stage I patients * 18% in stage II patients * 31% in stage III patients -11.6% of CTC negative patients developed recurrence compared to 21.1% of CTC positive patients -RFS was 88.4% in CTC negative patients compared to 78.9% in CTC positive patients -BRD was 4.3% in CTC negative patients compared to 14.5% in CTC positive patients	[91]
CellSearch	Curable colorectal cancer (stages I–IV)	-Diagnosis -Prognosis	287	-Preoperative assessment of CTC -CTC threshold ≥1 CTCs/7.5 mL	OS and PFS	-CTC was detected more frequently in metastatic patients -CTC were not associated with clinicopathological variables in non-metastatic patients -in preoperative CTC detection: CTCs≥1/7.5 mL was associated significantly with worse OS (HR = 5.5) and PFS (HR = 12.7)	[92]
CellSearch	Resectable esophageal cancer	-Prognosis	100	-Preoperative assessment of CTC -CTC threshold ≥1 CTCs/7.5 mL	OS and RFS	-CTCs in 18% of patients -CTC positive patients had inferior OS (HR: 3.128) and RFS (HR: 5.063), holds in multivariate analysis	[93]
Epic (Epic Sciences)	Metastatic castration-resistant prostate cancer	-Prognosis	161	-New line of treatment at the first follow-up	OS, rPFS (r:radio)	-CTC negative patients have better OS and rPFS compared to CTC positive patients -all AR-V7-CTC positive patients were resistant to ARS inhibitors (63% of CTC-positive cases) -AR-V7-CTC positive patients treated with Taxane had favorable OS compared to those treated with ARS inhibitors (HR = 0.24)	[62]
EPISOT, CellSearch and flowcytometry preceded by enrichment using RosetteSep™ (STEMCELL technologies)	Head and neck squamous cell carcinoma	-Prognosis	65	-New line of chemotherapy and cetuximab -CTC was assessed at Day 0 (D0, before treatment), D7, & D21 -CTC threshold ≥1 CTCs/10–15 mL	PFS	-Pretreatment, CTC was detected with EPISOT, CellSearch and Flowcytometry in 69%, 21% and 11% of patients respectively. -at D0, the median PFS was 5.3 months for all the 65 patients with 0.08 probability of survival at 12 months. -PFS was significantly higher in patients with no CTC, or reduction between D0 and D7, compared to stable or increased CTC -CTC count on D21 was not significantly associated with PFS	[94]
RosetteSep™ (STEMCELL technologies) enrichment followed by flowcytometry	Metastatic colorectal cancer	-Prognosis	55	-CTC assessment after the first cycle of treatment -CTC threshold >30 CTCs/mL	OS and FPS	-CTCs were detected in all patients -CTCs>30 /mL associated with inferior OS and PFS, holds in multivariate analysis	[95]
CellSearch and DEPArray	Chemosensitive and chemo-refractory small-cell lung cancer	-Diagnosis -Prognosis	13	-CTCs from pretreated patients examined for copy-number aberrations (CNAs) using NGS (Illumina)	OS and PFS	-88 single CTCs were testes from 31 patients. -The classifier correctly assigned 83.3% of cases as either chemorefractory or chemo-sensitive. -Significant difference in PFS, but not OS, between chemorefractory and chemosensitive patients	[96]
ScreenCell^®^	Lung Cancer	-Diagnosis -Mutation Screening	93	-KRAS mutation assessment in CTC and cell tumor DNA (ctDNA) using COLD-PCR/HRM assay	KRAS mutation	-KRAS mutation was identified in 21.3% of patients -Mutation analysis in matched CTC DNA revealed 20 mutations in 23.2% of the patients -Mutation analysis in matched ctDNA samples revealed 26 mutations in 30.5% of the patients -Greater sensitivity and specificity for KRAS mutation detection in ctDNA than in CTCs	[97]
CellSearch	-Advanced Non-Small-Cell Lung Cancer	-Prognosis	41	-CTC threshold >1 CTC/7.5 mL -EGFR mutation analysis in a single-armed phase II clinical trial of erlotinib and pertuzumab using TaqMan Gene Expression Assay	PFS and FDG-PET tomographic scan	-CTCs were detected in 78% of patients -Greater sensitivity for EGFR mutation detection in ctDNA than in CTCs -Lower CTC count was associated with longer PFS	[98]
